# Implementing group research assignment in undergraduate medical curriculum; impact on students’ performance and satisfaction

**DOI:** 10.1186/s12909-020-02137-x

**Published:** 2020-07-20

**Authors:** Z. Alrefaie, A. Al-Hayani, M. Hassanien, A. Hegazy

**Affiliations:** 1 Physiology Department, Faculty of Medicine, King Abdulaziz University, Jeddah, Saudi Arabia; 2 Physiology Department, Faculty of Medicine, Cairo University, Cairo, Egypt; 3 Vice President, Educational affairs & Anatomy Department, Faculty of Medicine, King Abdulaziz University, Jeddah, Saudi Arabia; 4 Educational affairs and College of Pharmacy, King Abdulaziz University, Jeddah, Saudi Arabia; 5 Medical Biochemistry department, Faculty of Medicine, Tanta University, Tanta, Egypt; 6 Community Medicine, Faculty of Medicine, King Abdulaziz University, Jeddah, Saudi Arabia

**Keywords:** Medical curriculum, Undergraduate, Research skills, Communication skills

## Abstract

**Background:**

Medical educators need to integrate research skills within undergraduate medical curriculum to help students perceive their relevance to routine doctor’s practice. The current work aimed to assess the impact of including group research assignment in the endocrine module to third year medical students on attaining some research, communication and E learning skills and on their performance in the module.

**Methods:**

Students carried out a group research activity (*N* = 10), wrote a report and presented their work as a poster, booklet or video clip. Multiple evaluation methods were used; a questionnaire to assess students’ satisfaction and perception towards the skills acquired and a rubric to grade the research report and presentation. Also, students’ final grades in the module were compared with that of the previous cohort who didn’t conduct the research assignment.

**Results:**

Students’ response rate to the questionnaire was 50%. 73.6% of students agreed that research enhanced critical evaluation of literature while 65.5% felt confident to further participate in research and 66.7% were satisfied about the whole research experience. Mean score of assignment was 84% for female students and 78% for male students.

Grades of the current cohort in the endocrine module were significantly higher than that of the preceding cohort (78.7 ± 11 and 70.2 ± 13 respectively *P*< 0.001).

**Conclusion:**

The current study pointed to the positive impact of implementing group research assignment within the undergraduate medical curriculum. Students were satisfied about the research exposure, agreed attaining some skills and got higher grades than preceding peers.

## Background

Competency-based medical education aims to foster attaining certain levels of determined competencies by medical students and graduates. It includes but not limited to; critical thinking, evaluating evidence, research and communication /interpersonal skills [1]. According to [2], developing research skills would enable the use of cognitive flexibility theory which underlies the application of evidence-based approach in medical career (3). According to [3, 4], developing research skills is central to medical school education and including research skills within undergraduate medical curriculum is crucial. Moreover, the results of MEDINE2 Erasmus Thematic Network for Medical Education in Europe 2009–2013, strongly suggested that learning outcomes related both to ‘using research’ and ‘doing research’ should be core components of medical curricula in Europe [5].

As a member in the pre-clinical phase curriculum committee and a director of the endocrine module together with other colleagues, we thought to introduce third year students to basic concepts and skills of research and team work though a group research task that is related to the module’s learning outcomes. The current work aimed to assess the impact of this research assignment on attaining research, communication and E learning skills and the impact on students’ overall performance in the module by comparing student’s final grades to that of the preceding cohort who weren’t exposed to the research task.

## Methods

### Study design

The current cross-sectional comparative study was approved by the module committee, curriculum committee and the vice dean for the pre-clinical phase and ethical approval was obtained from the Research Ethics Committee, Faculty of Medicine, King Abdulaziz University.

Students’ enrollment was the sampling technique as all third-year students registered in the endocrine module on 2018/2019 academic year participated in the study.

#### Planning

1. List of research topics was discussed and approved by the module committee over 3 consecutive meetings. All topics were related to obesity and diabetes mellitus from different perspectives
2. Orientation of faculty regarding design and use of rubrics was accomplished through a workshop that was presented by the medical education department and all committee members were invited and rewarded with attendance certificates. Three rehearsal committee meetings were set later.
3. Students’ orientation was conveyed through detailed description of the research activity in the study guide including; objectives of the activity, list of topics, assessment technique and the rubric used, required submissions, how to communicate with mentors and the submission deadline. Further orientation was offered during the overview session in beginning of the module and the assignment slot in BlackBoard (BB, college E learning management system).
4. Five-point Likert’s scale questionnaire was adopted from the National Center for Vocational Education Research NCVER Student Outcomes Survey 2010 questionnaire. Some changes were made to the original questionnaire to suit the current objectives and an expert was consulted to review it. After being piloted with a representative group of students, minor modifications were further made and subjected to check and approval by the same expert. The Cronbach alpha coefficient for the modified questionnaire was 0.95 reflecting excellent internal consistency. The questionnaire consisted of 12 closed-ended items reflecting attitude towards gained skills; research skills (4 items Nu 1-4), communication/interpersonal skills (3 items Nu 5-7), E learning skills (3 items Nu 8-10) and overall satisfaction (2 items Nu 11 & 12).
5. A rubric for grading the research assignment was revised and approved by the committee and a member from the medical education department.

#### Implementation

All male and female students of 2019 cohort were classified into groups (*N* = 10 within the group), and each group was given a research topic and assigned a member of the committee as a mentor. There were 46 groups; 24 groups for female students and 22 for male students. Each topic was carried out by a female group and a male group who worked separately, and all submissions were checked for plagiarism through the SafeAssign option of the BB.

The BB was used to; divide students anonymously into groups, upload all instructions and rubric and set discussion boards for the communication between mentors and students. Also for the students to check for plagiarism, upload reports and presentation forms.

Two meetings were held between each group and the mentor. Meetings and discussion boards aimed to:

- Approving students’ formulated objectives
- Guide the literature search and report writing
- Guide the preparation of the presentation form

#### Post-implementation

**Fig. 1:**
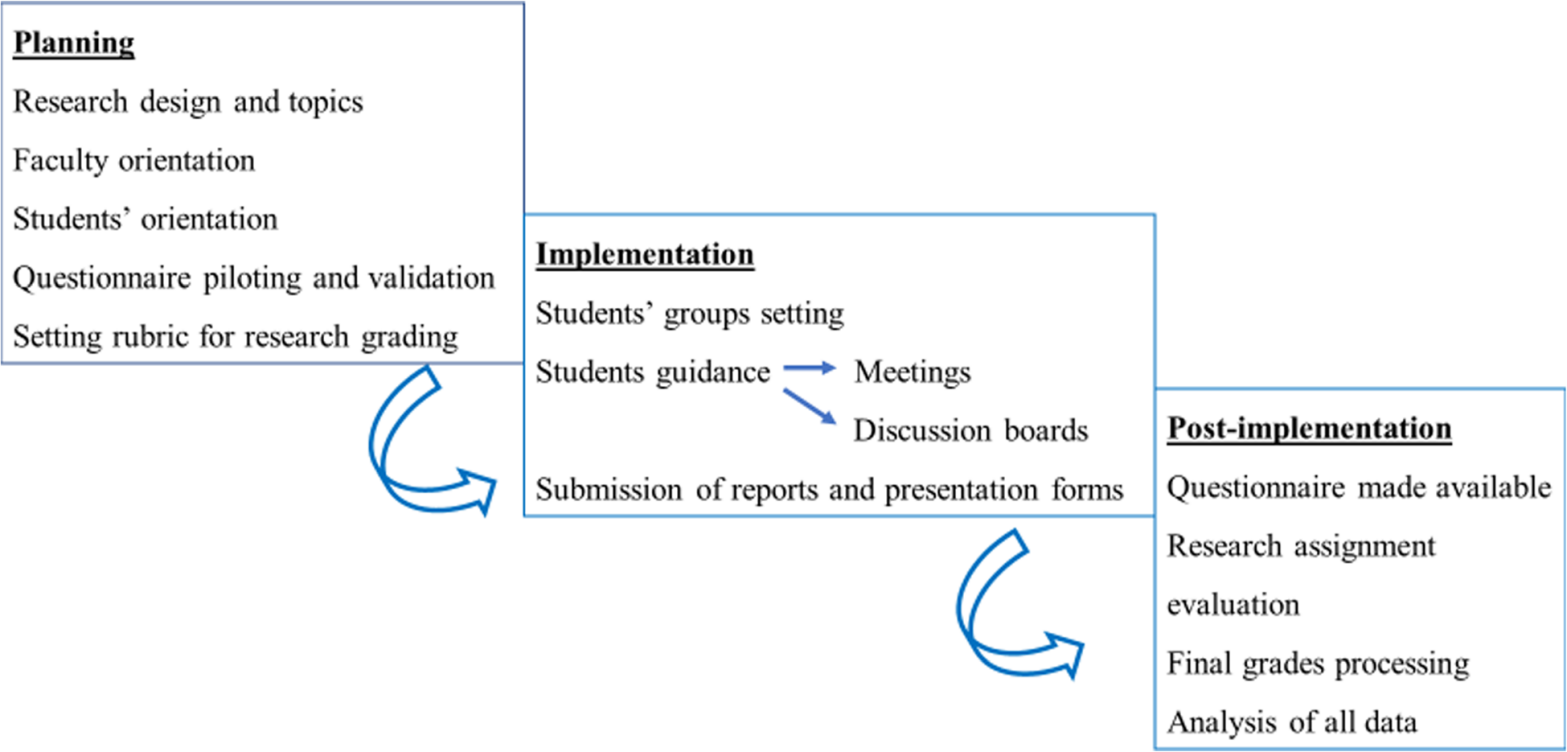
Illustration of different phases of implementation

Students were allowed a week after the final exam to submit the report and the presentation. The link to questionnaire was sent to students following the submission to assess their attitude and satisfaction regarding attained skills besides to overall satisfaction. Taking the questionnaire was optional and considered as the students’ consent to participate. Questionnaire was made available for 1 week as students became busy with the following module. Evaluation of the research assignments was carried out according to the rubric and research scores together with the final module grades were gathered Fig. 1.

#### Statistical analysis

The data were entered and analyzed using the Statistical package for the Social Sciences, SPSS 20 (SPSS Inc. Chicago, Illinois, USA). Mean and standard deviation were given for quantitative variables while frequencies and percentages were given for categorical variables. Independent sample t- test was used to compare the module final grades between 2018/2019 and 2017/2018 cohorts. The level of significance was set at *P* < 0.05.

Multiple regression analysis was used to detect the predictors (whether gained research skills, communication skills or electronic learning skills) that influenced the research score. Responses to each item in the Likert’s scale were transformed into score; if the answer was strongly agree / agree, it is given 3. If the answer was neither agree/ disagree it is given 2, and if the answer was strongly disagree / disagree it is given 1. Then the score for each item in the questionnaire was calculated and the mean of the scores for each group of items was also calculated and used for the regression analysis.

## Results

**Fig. 2:**
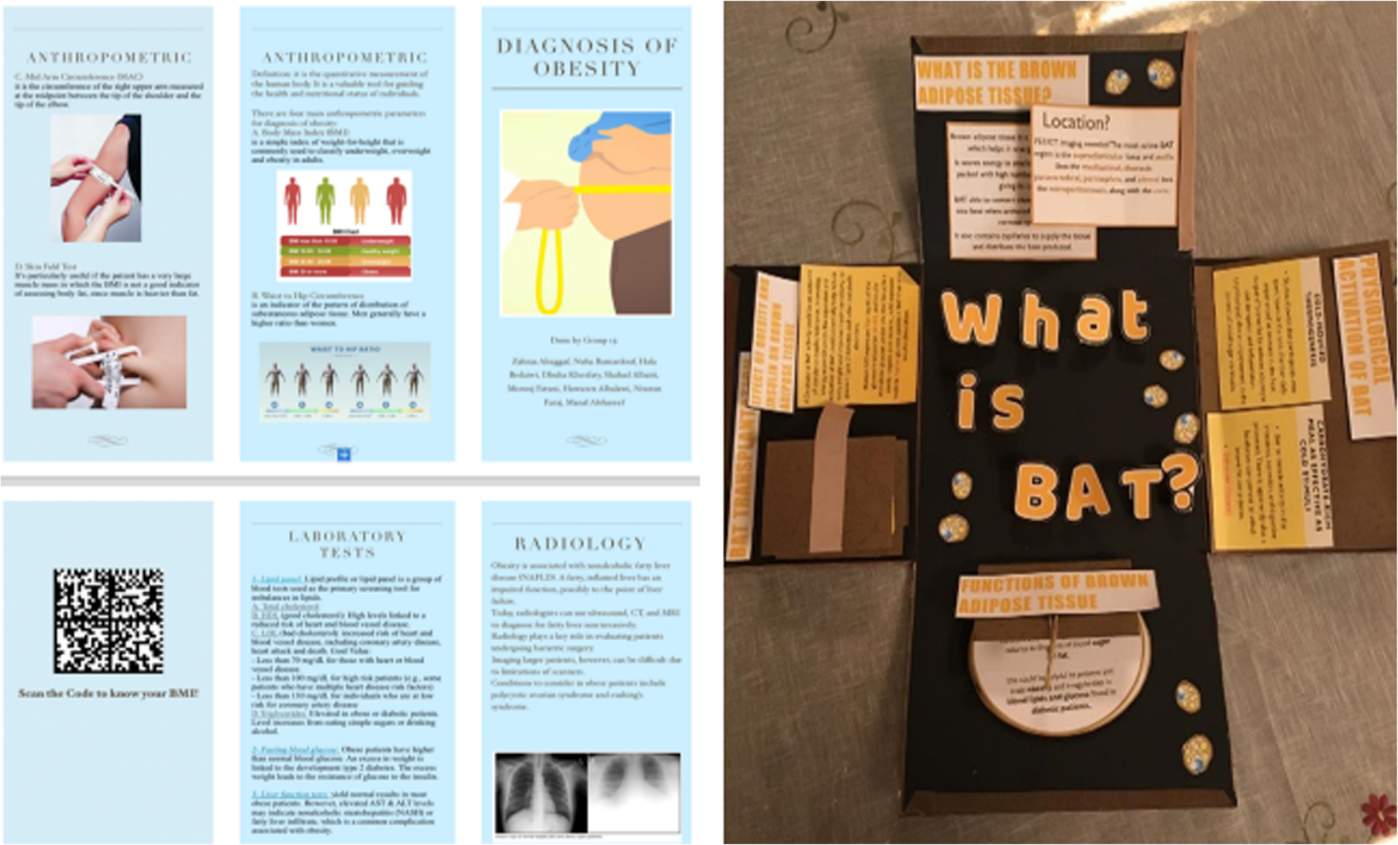
Examples of students’ presentation of research assignments; a brochure and cartoon model

The Final grade of the module is out of 100; 5 marks were allocated for the research assignment (research score) while the remaining 95 marks were distributed over the written assessments (including both MCQs and case scenarios with MEQs {modified essay questions}), practical exam (OSPE) and problem based sessions (PBL) and student prepared presentation (SPP) Fig. 2.

A structured rubric was used in the current study to evaluate students’ performance in the research assignment, the mean for students’ score in research was 78 and 84% for male and female students respectively and 81% for the whole cohort (Table 1).

**Table 1:** Study groups’ numbers, final grades and research score for batch 2019

	N	Minimum	Maximum	Mean	Std. Deviation
Female grade 2019	240	39.00	96.00	80.71	10.28
Male grade 2019	226	36.00	99.00	76.67	11.86
Total grade 2019	466	36.00	99.00	78.72	11.25
Male grade 2018	198	11.00	91.40	66.74	14.91
Female grade 2018	208	18.75	90.22	73.42	11.10
Total grade 2018	406	11.00	91.40	70.16	13.50
Total research grade	466	0.00	5.00	4.08	.87

**Fig. 3:**
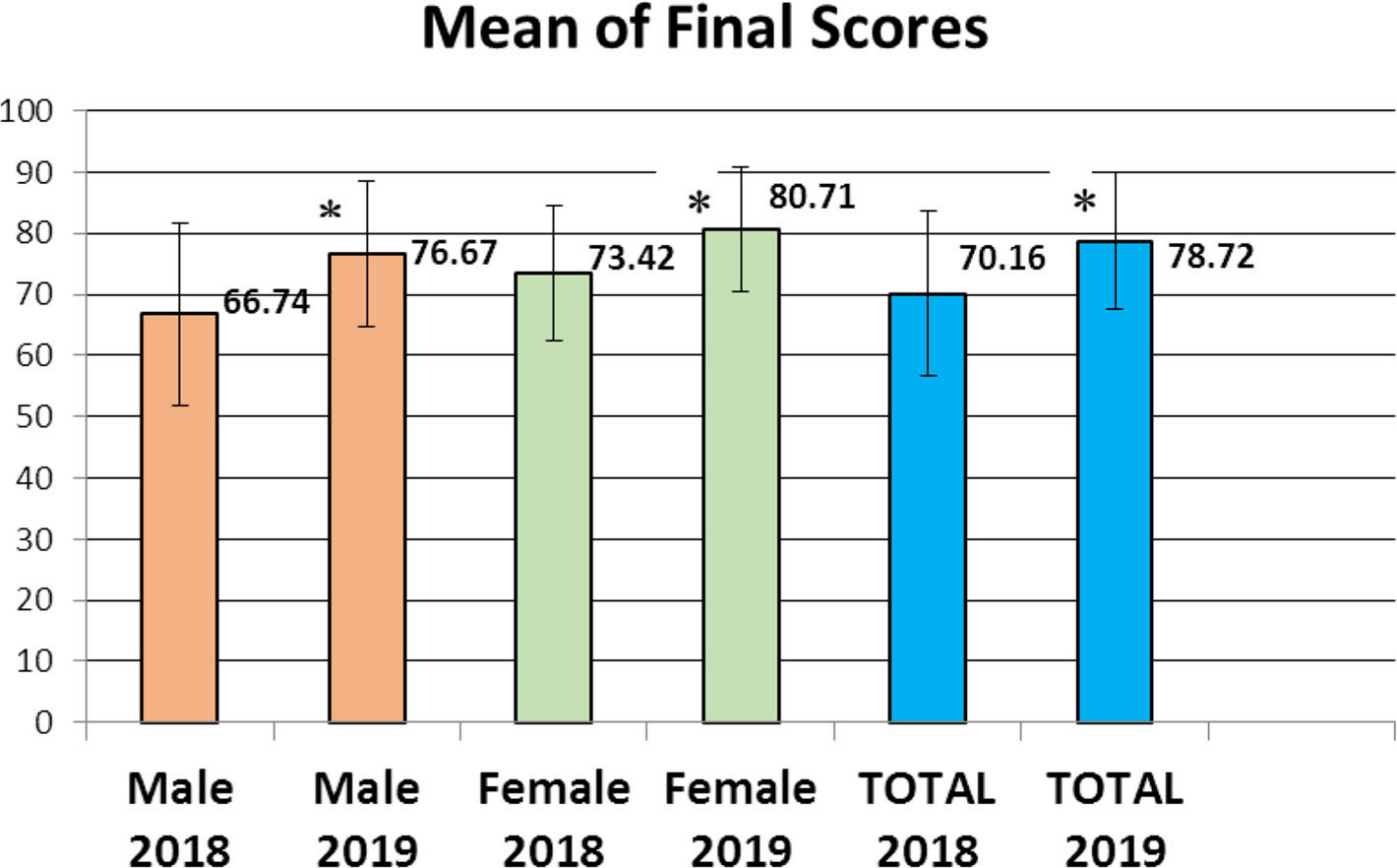
Final grades of male, female and total student of 2018 & 2019 cohorts

The means ± SD for final grades of the current cohort who carried out the research task and the preceding cohorts who did not are shown in Fig. 3, with the current cohort’s grades being significantly higher (*P* < 0.001).

The number of students who responded to the questionnaire was 229 out of 2019 cohort (466) with a response rate of about 50%, this is probably because students became busy with the following module and many students feel saturated regarding questionnaires, as they have to complete 4 different questionnaire sent by the quality unit at the end of each module. In addition, the questionnaire was made available for only 1 week to avoid interference with the next module.

The 229 responses comprised 127 and 102 from female and male students respectively.

Students’ response to the questionnaire is shown in Table 2, as the percentage of respondents who agree, disagree, neither agree/disagree to different items. The satisfaction about enhancement of critical evaluation of literature got the highest agreement (73.6%). 65.5% of students agreed feeling confident to further participate in research while 66.7% felt satisfied about the whole research experience.

**Table 2:** Response to questionnaire; percentage of respondents (229) who strongly agree/agree, neither agree/disagree or strongly disagree/disagree to different items

Item	Strongly Agree/ Agree %	Neither Agree/ Disagree %	Strongly Disagree/ Disagree %
1- The research improved my internet search skills	61.5	13.8	24.7
2- The research enhanced my evaluation of the related papers (critical appraisal)	73.6	16.1	10.3
3- The research helped me to write a review about certain topic	63.8	16.1	20.1
4-The research topic added to my knowledge in endocrine system	64.4	12.6	23
5- The research improved my verbal communication skills with colleagues	60.9	17.8	21.3
6- The research helped me develop my ability to work as a team member	67.8	14.9	17.2
7- The research assignment helped me to plan and distribute work among members	67.2	13.2	19.5
8- I received useful feedback in response to my questions (through the discussion board)	69.5	16.1	14.4
9-I successfully utilized SafeAsign (for plagiarism) using the BlackBoard	67.9	19.7	12.4
10- Dealing with the BlackBoard was easy	64.4	12.6	23
11- Following this experience, I feel more confident about participating in research projects	65.5	14.4	20.1
12- Overall, I was satisfied with the quality of this training	66.7	13.2	20.1

Regression analysis was performed to detect the predictors that influenced research score (Table 3). It demonstrates that the most common predictors for research grade were research skills and E learning skills. It shows that students who had felt satisfied about acquiring research skills are more likely to get good research score (adjusted Odd’s Ratio AOR = 2.8), followed by students who were satisfied about gaining E learning skills (AOR = 2.1).

**Table 3:** Predictors of the research score among the participants, logistic regression analysis

	B	Sig.	AOR	95% CI	
Research Skills Score	1.03	0.15	2.81	0.67	11.70
Electronic Learning Skills Score	**0.76**	**0.17**	**2.14**	**0.70**	**6.51**
Communication/Interpersonal Skills Score	**−1.02**	**0.09**	**0.35**	**0.10**	**1.21**
Satisfaction Score	**−0.28**	**0.62**	**0.75**	**0.23**	**2.35**

## Discussion

Skills and experiences are attracting great attention in all educational programs. Most of the medical frameworks like Tomorrow’s Doctors and ACGME encompass a lot of skills besides to knowledge. Application of the concept of evidence-based medicine requires students to be able to search for and critically appraise literature. Moreover, effective communication and team working are ranked as highly important by health professionals [6] and by medical students themselves [7].

Many learning activities can enhance research related attributes and research project is a strategy that can develop variety of skills including analytical, communication besides to research skills [8].

The current work aimed to provide third year medical students with a group research opportunity through presenting a report over a topic related to endocrine module objectives. Evaluation of the implemented strategy revealed students’ satisfactory attitude regarding skills developed during the project and enthusiasm to further participate in research. In addition, research scores reflected above average performance specially for female students, while the final students’ grades in the endocrine module were significantly higher when compared to their preceding peers who didn’t have a research assignment. Comments on this research activity submitted by students to the FOM KAU quality unit were quite encouraging like “I enjoyed the research project and learned new things despite time constraint”. Moreover, students performed well in the endocrine related questions compared to other courses and modules in the progress exam implemented at the end of the 2018–2019 academic year. The results of this exam were officially reported in the first curriculum committee meeting on November 2019.

One of the reasons that might explain students’ overall performance improvement over the preceding cohort could be the passion and interest in the course that helped consolidation of gained information. This passion was revealed in students’ reflections on their roles and teammates as part of the submission. Many students just listed their part of the task and their point of view concerning others’ participation, while some other comments unveiled the positive energy felt by students like; “The teamwork was very great, everyone was so excited, they were very supportive, creative and they made me enjoy the work. Best team I ever worked with and if I had to do it again, I would.”

“Everyone did an excellent job without any delay. I’m really blessed for being with this group”.

“I had a quite good experience with the group, we have been sharing information between each other so, that gives us a good understanding of the topic.”

“I think my group was very cooperative in this Project. We all worked together to choose the design for the poster, the information that was added and even the colors. We didn’t have any arguments or troubles; our decisions were based on the benefit of this project. Overall, it was a very educational and fun experience.”

“I enjoyed working with my group in this research, it was amazing.”

Moreover, the increased students’ grades relative to the preceding cohort who didn’t have a research task may be attributed to the further readings within the research topics that were related to the module learning outcomes. One other thing that might partially explain the higher final grade of 2019 cohort is the mean score for the research task that was 4.1/5, it probably helped in raising the mean of their final grades.

The current work introduced third year medical students to some basic research skills like; formulating objectives, searching for relevant valid literature, writing a report and furthermore working as a team to accomplish their task. It was the first instance for those cohort to go through such experience that showed promising results and was acknowledged by our college administration. What seemed encouraging that our model was partially replicated by colleagues in the committee of neuroscience module who included in their course a group poster submission on some neuroscience related topics.

Recently, [9] enrolled fourth-year medical students attending pathology course into groups that were given different cases and asked to submit an abstract together with a poster on these cases. Students were judged with a five-point scale rubric for both abstract and poster by trained faculty who reported that research activity seemed achievable and interaction with students was attractive. The way Rojas et al. introduced students to research is like ours considering the implementation within the context of a particular course and within a relatively limited time frame. In favor of our work over that of Rojas et al., is our evaluation of students’ perception and satisfaction towards the research experience in addition to assessing the impact of the research experience on final grades of the course.

Cain et al [10] described the medical student summer research program implemented in University of Texas, medical school and the results of two surveys conducted to obtain students’ views about this program. The summer research course ran over 8 weeks following successful completion of the first year, where faculty accommodated students in their laboratories and completed a pass/fail form for students at the end of the program. A pre and post-surveys were completed by students and showed that around 96% of students strongly agreed that research was vital to the future and about 75% felt that research is integral to be a physician while 63% strongly disagreed that they would devote most of their career to research.

From the results of Cain et al. together with ours, we can assume that whether students would pursue a research career or not, they still perceive the value of being offered a research experience to their future.

An example of summer research programs was evaluated in the work published by [3] where medical students participated in summer research six-weeks program in family medicine. The authors revealed that the research experience was well perceived by the students who demonstrated sustained productivity year after year.

Summer research projects could offer plentiful time and less stressful experiment depending on the design and outcomes, but the current work aimed not only to introduce students to the basic research skills but also to include the research project within the context of the module and in alignment with its objectives to further consolidate the knowledge in addition to the acquired skills.

Up to our knowledge, the study of [11], is the only one that assessed undergraduate medical students’ participation in research in FOM KAU. It was conducted to assess the publication practices of medical interns who graduated from FOM KAU. 31% of the responding interns started research, upon personal communication with faculty, during their undergraduate study years. About 12% discontinued their research, whereas only 7% submitted their research for publication. Respondents showed positive attitude towards research, however they considered lack of time and training as the main obstacles for conducting and publishing research.

## Conclusion

The current work showed that research assignment carried out by third year medical students during the endocrine module in topics that lie within the module’s themes enhanced many students’ skills as perceived by students, significantly increased students’ overall achievement in the module and pursued students’ enthusiasm to further participate in research.

In addition, enhancing faculty research appraisal skills and awareness of including research practices in undergraduate medical curriculum were among the deliverables achieved by the current work.

We feel enthusiastic towards sustainability of this experience and are planning for the upcoming cycle of implementation to involve display of students’ presentations of their research topics in “Endocrine Day” to further disseminate the idea and share experiences among students.

### Limitations

Relatively low response rate to the questionnaire, consequently we will reconsider the timing and availability of the questionnaire, and probably participation will remain optional to target students who honestly want to reflect upon their experience.

Moreover, focus groups will be also implemented to obtain more qualitative aspects of the students’ perception.

## Data Availability

The datasets used and/or analysed during the current study are available from the corresponding author on reasonable request.

## Abbreviations

BB: BlackBoard; electronic learning management system in our institution
FOMKAU: Faculty of Medicine, King Abdulaziz University
